# Direct Incorporation of Expert Opinion into Parametric Survival Models to Inform Survival Extrapolation

**DOI:** 10.1177/0272989X221150212

**Published:** 2023-01-16

**Authors:** Philip Cooney, Arthur White

**Affiliations:** School of Computer Science and Statistics, O’Reilly Institute, Trinity College Dublin, Dublin 2, Ireland; School of Computer Science and Statistics, O’Reilly Institute, Trinity College Dublin, Dublin 2, Ireland

**Keywords:** survival models, extrapolation, expert opinion

## Abstract

**Background:**

In decision modeling with time-to-event data, there are a variety of parametric models that can be used to extrapolate the survival function. Each model implies a different hazard function, and in situations in which there is moderate censoring, this can result in quite different survival projections. External information such as expert opinion on long-term survival can more accurately characterize the uncertainty in these extrapolations.

**Objective:**

We present a general and easily implementable approach to incorporate various types of expert opinions into parametric survival models, focusing on opinions about survival at various landmark time points.

**Methods:**

Expert opinion is incorporated into parametric survival models using Bayesian and frequentist approaches. In the Bayesian method, expert opinion is included through a loss function and in the frequentist approach by penalizing the likelihood function, although in both cases the core approach is the same. The issue of aggregating multiple expert opinions is also considered.

**Results:**

We apply this method to data from a leukemia trial and use previously elicited expert opinion on survival probabilities for that particular trial population at years 4 and 5 to inform our analysis. We take a robust approach to modeling expert opinion by using pooled distributions and fit a broad class of parametric models to the data. We also assess statistical goodness of fit of the models to both the observed data and expert opinion.

**Conclusions:**

Expert opinions can be implemented in a straightforward manner using this novel approach; however, more work is required on the correct elicitation of these quantities.

**Highlights:**

- Presentation of a novel and open-source method to incorporate expert opinion into decision modeling.
- Extends upon earlier work in that expert opinion can be incorporated into a wide range of parametric models.
- Provides methodological guidance for directly including expert opinion in decision modeling, which is a research focus area in NICE TSD 21.^1^

The primary aim of many studies is to analyze the time until a prespecified event of interest occurs. In this setting, the response variable is the time until the event occurs, which is often called failure time, survival time, or event time. Time-to-event data are usually not observed for all observations under study, primarily because the data from a study are analyzed at a point in time at which some individuals have not yet experienced the event, resulting in these observations being censored. In multiarm clinical trials that have a time-to-event outcome, the primary objective is usually to identify whether there is a statistically significant difference in the expected survival times of the treatment arm relative to a control arm. In health technology assessments, the primary focus is to assess the long-term expected survival of the treatment groups so that the incremental health outcome of an intervention can be calculated. Except in situations in which we are willing to assume that the long-term difference in health outcomes is similar to the differences in survival observed during the trial,^2^ we are required to assume a parametric form for the data. These parametric models extrapolate the observed survival data to make long-term survival projections that are crucial to cost-effectiveness decision making.

Differences in long-term predictions can be particularly pronounced when a high proportion of the survival times are censored and may produce clinically implausible survival estimates.^3^ A number of solutions have been proposed for this issue, including model averaging, using external data, and incorporating expert opinion.^4–6^ When expert clinical opinion is available, it is important to use this information in the modeling process. These opinions are often not integrated in a formal way, with survival models typically estimated using maximum likelihood (i.e., based on the data alone) before choosing the parametric model for which expected survival appears to be compatible with the expert opinion. This approach has a number of weaknesses. Primarily, it is difficult to identify the most appropriate model if several models appear consistent with the expert opinion. In the opposite scenario, when none of the models meet the expert’s criteria, the best choice of model is again unclear. It would be preferable to have a measure of statistical fit that takes account of the degree of agreement with the expert opinion as well as the observed data, rather than making a decision based solely on whether the predicted quantity from the model is within the expert’s plausible range.

In this article, we consider how long-term survival estimates provided by clinical expert opinion can be directly incorporated into the model estimation procedure. We do so by adopting a framework in which the expert opinion that has been elicited on the observable data space is used to modify the density of the parameter space. Our approach is compatible with the SHELF elicitation framework, including when multiple expert opinions are available,^7^ and can be applied to many parametric models, from the exponential distribution, which assumes a constant hazard, to spline models that can accommodate bathtub type hazards. This approach generalizes previous work,^5^ in that we consider the parametric survival models commonly used in decision making, evaluate model fit based on goodness of fit to both data and expert opinion, and do not restrict expert opinion to be represented by a single normal distribution.

The rest of the article is organized as follows. We provide a review of some methods that incorporate expert opinion into parametric survival models. Subsequently, we introduce the proposed statistical method and discuss considerations when aggregating the opinions of multiple experts. We then present an application of the method whereby the survival times of pediatric acute lymphoblastic leukemia patients treated with tisagenlecleucel are integrated with expert opinions about survival at various time points.^8,9^ We conclude the article with a discussion of its key ideas along with a summary of the challenges involved in eliciting expert opinion. The supplementary material contains four appendices which provide more detail on the method described here. In Appendix A, we validate our approach with a previously published example whereby expert opinion on median life was incorporated into the survival function. In Appendix B, we describe some technical details regarding the estimation of certain parametric models. In Appendix C, we perform a simulation study to assess the effect of priors on posterior survival extrapolations when including expert opinion, and in Appendix D, we perform a simulation study to assess the impact of bias in expert opinion on extrapolated survival. All methods outlined in this article are available for use as an R package called expertsurv at https://github.com/philip-cooney/expertsurv.^10^

## Previous Literature

Much of the initial work on this topic is from reliability analysis, with Weibull models incorporating expert opinion about the median survival using chi-squared or normal priors.^11,12^ One disadvantage of both approaches is that the experts are also required to think about the mean and variance of the shape parameter of the Weibull distribution (i.e., parameter space), which is much more difficult than eliciting information in the observable data space. Other work estimated the Weibull model based on expert opinion from either the mean, mode, and quantiles of survival time and a hyperparameter representing the effective sample size of the opinion, avoiding the need to elicit expert opinion on the parameter space.^13^

Another approach uses hyperparameters to incorporate expert opinion for survival models for which conjugate priors or priors with the same form as the likelihood exist (exponential, gamma, and Weibull). For 2-parameter models, however, this approach requires the assumption that one of the parameters is already known.^14^ The approach generates informative priors by calculating their hyperparameters using sufficient statistics such as (but not limited to) the number of events, censored observations, and the sum of event times.

Survival models with covariates can also incorporate expert opinion, whereby the expert contributes a distribution conditional on the values of the covariates at a design point. Using a class of priors referred to as data augmentation priors in which the prior has the same form as the likelihood, expert opinion was incorporated at different levels of a covariate for exponential and log-normal examples, again through deriving hyperparameters.^15,16^

In the context of health technology assessment, Ouwens^17^ incorporated expert opinion about survival probabilities at a particular time point for 1- and 2-parameter models by reexpressing one of the parameters as a function of the survival probability at the chosen time point and the other parameter (if applicable). This approach considers a broader family of parametric models than those previously described. The approach samples both a survival probability from the expert’s prior distribution and the second parameter from its (noninformative) prior and uses these to calculate the first parameter. A similar hierarchical Bayesian approach (although from the field of ecology) considered the Weibull model with expert opinion elicited on mean survival at different covariate levels for multiple experts.^18^

Cope et al.^5^ introduced a method to incorporate expert information regarding survival probabilities when it has been provided at multiple time points. A Bayesian approach is used to fit a hazard function to the observed data and the hazards implied by the long-term survival beliefs of the expert. Weibull, Gompertz, first- and second-order fractional polynomials can be fit with this approach using the JAGS statistical program^19^; however, it is not clear if the expert opinion modifies the model parameters or if it is solely the hazards implied by the expert’s survival beliefs that are used to extrapolate the survival beyond the observed data.

In this article, we consider how to incorporate multiple experts’ opinions about survival probabilities at multiple time points into an analysis of time-to-event data. As noted above, limitations of previous approaches include that they elicited beliefs on the parameter space (or assumed the parameters are known)^11,12,14^ or incorporated opinions using hyperparameters that are specific to one type of parametric model,^15,16^ while even the most general approaches do not include all of the standard parametric models typically used in decision making.^5,17^ The approach described in this article is more general than previous approaches, as it can in principle be applied to any quantity for which an expert can provide an opinion, for example, median or mean survival times (which may be at different covariate levels) and is compatible with a wide range of parametric survival models.^a^ In addition, standard goodness-of-fit measures can be used with the method to identify the parametric model that has the best fit to both the expert opinion and the data.

## Survival Analysis with Expert Information

Suppose there are *n* subjects under study and that associated with each individual i is a survival time $t_{i}$ and a fixed censoring time $c_{i}$. Each $t_{i}$ is assumed to be independent and identically distributed (i.i.d) with density f(t) and survival function S(t). The exact survival time of an individual will be observed only if $t_{i}\leq c_{i}$ and if not $t_{i}=c_{i}$, with the status indicated by

$$v_{i}=\left\{ \begin{array}{c} 1,\;t_{i}\leq c_{i}\\ 0,\;t_{i}>c_{i} \end{array}\right..$$

For a parametric survival model, with an associated set of parameters $\theta ={\left(\theta_{1},\ldots ,\theta_{p}\right)}^{T}$, the likelihood function given the observed data D is $L\left(\theta |D\right)=\overset{n}{\underset{i=1}{\Pi }}f{\left(t_{i}\right)}^{v_{i}}S{\left(t_{i}\right)}^{1-v_{i}}=\overset{n}{\underset{i=1}{\Pi }}h{\left(t_{i}\right)}^{v_{i}}S\left(t_{i}\right)$, where h(t)=f(t)/S(t) denotes the hazard function. In a Bayesian analysis, we assume a prior distribution for θ denoted by π(θ). The posterior distribution is then π(θ|D)∝L(θ|D)π(θ).

As a running example, consider an exponential distribution, with associated hazard h(t)=θ and survival function S(t)=exp{−θt}. The likelihood of an exponential model is then $L\left(\theta |D\right)=\theta^{\sum_{i=1}^{n}v_{i}}exp\{-\theta \sum_{i=1}^{n}t_{i}\}$. If the prior distribution for θ has been specified as G(α,β), that is, a gamma density with parameters α and β, then the posterior distribution is available in closed form as a gamma distribution $G(\alpha +\sum_{i=1}^{n}v_{i},\beta +\sum_{i=1}^{n}t_{i})$. While in this case the posterior distribution is tractable, the Bayesian inference for other distributions is more challenging and relies on modern computational methods for inference. In this article and in the associated R package, we consider the exponential, Weibull, gamma, Gompertz, log-normal, log-logistic, generalized gamma, and Royston-Parmar models using standard parameterizations (some further details regarding Gompertz and generalized gamma models are presented in Appendix B).^20^

### Integrating Expert Opinion with Trial Data

Consider the situation in which an expert has an opinion about the survival probabilities at potentially multiple times $t^{*}=t_{1}^{*},\ldots ,t_{k}^{*}$. In other words, expert opinion has been elicited on the observable data space rather than the parameter space. We propose incorporating this information into the analysis by expressing the elicited quantity in terms of parameters φ, which will contribute a “loss” or penalization of the likelihood based on the discrepancy between the survival estimated by the model parameters θ and the elicited opinion.^21^ The parameters φ will typically be parameters of a specific probability distribution describing the expert’s opinion about the survival at each of the times $t^{*}.$ In this approach, the model parameters will be estimated based on their fidelity to both the data and expert opinion, with the relative strength determined by the number of observations and precision of the elicited belief.

In the most general situation in which we have *k* time points at which we wish to include expert opinion, we let $\varphi_{i}$ represent parameters associated with the time point *i* and $\pi_{t_{i}^{*}}(\theta |\varphi_{i})$ a loss function encoding expert opinion at time point *i*. The posterior distribution of the model parameters including expert opinion is

$$\pi \left(\theta |D,\varphi \right)\propto L(\theta |D)\overset{k}{\underset{i=1}{\Pi }}\pi_{t_{i}^{*}}(\theta |\varphi_{i})\pi (\theta ).$$

To fix this idea, consider an exponential model fit to data, with a normal distribution with mean $\mu_{expert}$ and variance $\sigma_{expert}^{2}$ (i.e., the parameters φ governing the loss function) describing the expert’s belief about survival at one particular time point $t^{*}$(suppressing the index *i*), so that $S(t^{*})\sim N(\mu_{expert},\sigma_{expert}^{2})$. The posterior density is then proportional to

$$\pi (\theta |D,\mu_{expert},\sigma_{expert}^{2})\propto L(\theta |D)\pi_{t^{*}}(\theta |\mu_{expert},\sigma_{expert}^{2})\pi (\theta ),$$

where

$$\pi_{t^{*}}(\theta |\mu_{expert},\sigma_{expert}^{2})$$

encodes the loss function for the expert opinion regarding survival at time t^*^ and π(θ) denotes a standard, typically weakly informative, prior for θ. For an exponential model, the survival at the elicited time point is, $S\left(t^{*}\right)=exp\left\{-\theta t^{*}\right\}$ so that

$$\pi_{t^{*}}\left(\theta |\mu_{expert},\sigma_{expert}^{2}\right)\propto \exp\left\{-\frac{1}{2}{\left(\frac{exp\left(-\theta t^{*}\right)-\mu_{expert}}{\sigma_{expert}}\right)}^{2}\right\}.$$

While the resultant posterior does not have a closed form, this is not of practical importance when using modern computational Bayesian methods. More generally, the advantage of this approach is that it can be applied to a wide family of survival models, including those with 3 or more parameters. It is also straightforward to represent the elicited opinion as other probability distributions (e.g., beta distribution) as well as incorporate additional time points. The contribution of the expert opinion can be implemented in programs such as WinBUGS and JAGS (through the zeros trick) or Stan (by simply incrementing the log probability).

Theoretical justification for this approach is provided by Bissiri et al.,^21^ who show that a valid and coherent update of a prior belief distribution to a posterior can be made for parameters that are connected to observations through a loss function rather than the traditional likelihood function, which is recovered as a special case. Although we have presented this method in a Bayesian framework, it can also be motivated from a frequentist perspective as an example of a penalized likelihood method.^22^ In this framework, we impose additional constraints on the parameter space by modifying the likelihood so that it is a function of the observed data and a further penalty term that will pull or shrink the final estimates away from the maximum likelihood estimates, toward values of the parameters that are more compatible with the elicited predicted survival at the time point(s) t^*^ Model estimation with this approach can be achieved using standard optimization techniques.^23^

When considering the analysis in a Bayesian framework, it is worth discussing the potential effect of the prior on the parameters π(θ) and the loss function that encodes the expert’s opinion $\overset{k}{\underset{i=1}{\Pi }}\pi_{t_{i}^{*}}(\theta |\varphi_{i}).$ Under this formulation, the information encoded in the loss function is distinct from the prior; however, it is possible that a particularly informative prior on the model parameters could also imply a density for survival at the designated time points, which conflicts with the expert’s opinion. We conducted an extensive simulation study, with various sample sizes and expert opinions (in terms of location and spread of the beliefs), for the parametric models we have implemented. We compared the results of the models’ fit using uniform priors for all parameters to relatively vague normal and gamma priors, which were more informative than those typically used in Bayesian analysis. Across the scenarios specified in the simulation study, the posterior distribution of the survival functions was effectively identical. In addition, we compared the results versus those obtained through penalized maximum likelihood estimation so that we could compare with a method that does not include a prior. We again obtained very similar results. From this, we can conclude that reasonably noninformative priors for the parameters should not conflict with information provided by an expert. Further details regarding the simulation studies are presented in Appendix C.

### Incorporating Multiple Expert Opinions

In some situations, the opinions of multiple experts are available, and in general, groups tend to perform better than the average individual in elicitation exercises.^24^ Although it is sometimes reasonable to provide a decision maker with the elicited expert probability distributions separately, the range of which can be studied using sensitivity analysis, it is often necessary to combine the distributions together into a single analysis. In many cases, for example, a single distribution is needed for input into a larger model, and that model has other inputs with structural uncertainties, so that a full sensitivity analysis may not be feasible. This is particularly true when a specific survival model is used as an input for a cost-effectiveness model, in which case decision makers are typically making choices with respect to the parametric model in question, in addition to other structural assumptions. Considering this model choice appropriately for each expert can be burdensome and inflate the number of scenarios presented to the decision maker.

Combination, or aggregation, procedures are often dichotomized into mathematical and behavioral approaches, although in practice, aggregation might involve some aspects of each.^7^ One such mathematical technique is opinion pooling, in which a consensus distribution for π(θ) is obtained as some function of the distributions $\pi_{1}\left(\theta \right),\ldots ,\pi_{m}\left(\theta \right)$ elicited from each of the *m* individual experts. Behavioral aggregation approaches attempt to generate agreement among the experts by having them interact in some way. This interaction may be face to face or may involve exchanges of information without direct contact or have an impartial observer to facilitate discussion (e.g., the SHELF protocol). In either case, the consensus distribution is then used as the prior for the analysis.

For the purpose of this article, we will focus on opinion pooling (as we require it for the example in the subsequent section) noting that these methods are simpler to implement than behavioral approaches, although the distributions that result need not represent the opinions of any one person, let alone a consensus opinion from the group of experts. In addition, these methods have “coherency” issues, as highlighted below.

We first consider the logarithmic opinion pool, which is obtained by taking a weighted geometric mean of the distributions,

$$\pi \left(\theta \right)\propto \overset{m}{\underset{j=1}{\Pi }}\pi_{j}{\left(\theta \right)}^{w_{j}}$$

with weights specified such that $\sum_{j=1}^{m}w_{j}=1$. When the decision maker is equally confident in the abilities of all experts, it is common to choose $w_{j}=\frac{1}{m}$ for all *j*. The key advantage of this approach is that it is externally Bayesian. When new data are obtained, one could either update each expert’s distribution individually and then combine the resulting posterior distributions using logarithmic pooling or first combine the expert’s distributions and then update the consensus distribution. These will result in the same posterior distributions. Continuing our example and assuming an exponential distribution with constant hazard, if m experts have expressed their prior beliefs about θ as gamma priors $G\left(\alpha_{j},\beta_{j}\right),j=1,\ldots ,m$, the pooled prior is also a gamma distribution, $G\left(\sum_{j=1}^{m}w_{j}a_{j},\sum_{j=1}^{m}w_{j}\beta_{j}\right)$, and the resulting posterior distribution is then $G\left(\sum_{j=1}^{m}w_{j}a_{j}+\sum_{i=1}^{n}v_{i},\sum_{j=1}^{m}w_{j}\beta_{j}+\sum_{i=1}^{n}t_{i}\right).$ If we were to compute the posterior distribution using each expert prior separately and then compute the logarithmic opinion pool post hoc, it is evident that the same posterior distribution would be obtained.

Logarithmic pooling, however, does not satisfy the marginalization property. Suppose each expert is asked about mutually exclusive events, A and B. If C is the event “A or B,” then coherency demands that Pr(C)=Pr(A)+Pr(B). There are 2 ways to obtain a pooled probability for C. We can compute the probability by adding Pr(A) and Pr(B) from each expert and pool the resulting sums, or we can pool the elicited probabilities for A and B first and then add the pooled results. With a logarithmic opinion pool, these approaches will not yield the same probability; however, it should be noted that this issue does not affect the pooling of survival probabilities.

Another form of expert pooling is the linear opinion pool

$$\pi \left(\theta \right)=\sum_{j=1}^{m}w_{j}\pi_{j}\left(\theta \right),$$

which is the weighted arithmetic mean of the distributions. This approach is not externally Bayesian. Continuing our example, a weighted sum of gamma distributions is not a gamma distribution and is not available in an analytic form unless the rate parameters are equal.^25^ Linear pooling does satisfy this marginalization requirement; however, no aggregation function can simultaneously satisfy the marginalization and externally Bayesian properties.

O’Hagan et al.^7^ noted that when using logarithmic pooling, the decision maker regards as implausible any values of θ that are considered implausible by any single expert. The linear opinion pool, on the other hand, concentrates more in the area where the experts’ opinions overlap, but it does not rule out values of θ that are supported by only one expert, which may be the reason linear pooling is more commonly used.^7^

**Figure 1:**
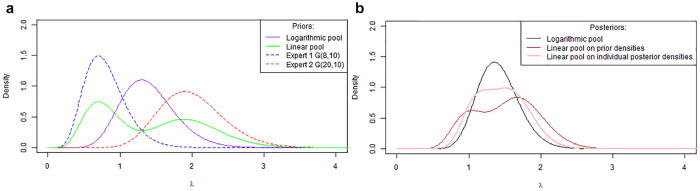
Aggregation of experts’ opinions (a) Linear and logarithm pooling of opinions; (b) Posterior distributions for linear and logarithm pooling.

We illustrate these properties in Figure 1, in which we consider a hypothetical example where 2 experts have provided their opinions on θ for an exponential model, with the experts holding somewhat conflicting opinions. We suppose expert 1 has a prior of G(8,10) (blue dashed line) and expert 2 has a prior of G(20,10) (red dashed line). Figure 1a presents both pooling approaches for the prior expert opinions, with the purple line representing the density of the opinion obtained by logarithmic pooling while the green refers to the density using linear pooling. As mentioned previously, the logarithmic pooling produces a pooled density that gives most weight to areas of overlap between the expert’s opinions, which peaks at the point where the density lines intersect. The linear pool is bimodal and retains the characteristics of the constituent prior distributions, with the 95% credible interval (0.40–2.79) being wider than that of the logarithmic pool (0.77–2.22).

Figure 1b shows the posterior distributions obtained when the individual expert’s priors and the logarithmic and linear pooled distributions are used as a prior for an exponential likelihood with the kernel of a G(10,7) distribution. For the logarithmic pooling, when we update each expert’s prior with data separately and then compute the logarithmic opinion pool of these posteriors, or pool both experts’ prior opinions and then update with data, we obtain the same posterior distribution (shown by the black line density). In contrast, linear pooling results in 2 separate posterior distributions depending on whether pooling was conducted on the individual priors (brown/maroon line) or individual posteriors (pink line). It is worth noting that the example presented here relates to the parameter space (i.e., experts gave opinion about the parameter); however, the results also hold when pooling on the opinions in the observable space.

More generally, when using our approach as described in the previous subsection and multiple expert opinions are available, the posterior has the form

$$\pi \left(\theta |D,\varphi \right)\propto L(\theta |D)\overset{k}{\underset{i=1}{\Pi }}\pi \left(\pi_{{t_{i,1}}^{*}}\left(\theta |\varphi_{i,1}\right),\ldots ,\pi_{{t_{i,m}}^{*}}\left(\theta |\varphi_{i,m}\right)\right)\pi (\theta )$$

where $\pi_{{t_{i,1}}^{*}}\left(\theta |\varphi_{i,1}\right),\ldots ,\pi_{{t_{i,m}}^{*}}\left(\theta |\varphi_{i,m}\right)$ denotes loss functions at time poing *i* for experts 1 through *m*, which are subject to the linear or logarithmic pool at each of the *k* time points. As noted previously, if we use a linear method to pool the expert information, then the resultant posterior will be different than if we ran separate analyses using each expert opinion and pooled the results a posteriori.

## Case Study: ELIANA Trial

Cope et al. elicited expert opinion on the expected survival probabilities at 2, 3, 4, and 5 y in pediatric patients with acute lymphoblastic leukemia treated with tisagenlecleucel, based on the available 1.5-y results from the ELIANA trial along with other available tisagenlecleucel data for pediatric acute lymphoma/leukemia.^5,8,26,27^ Elicitation was conducted in line with the SHELF methodology, in which for each time point, experts were asked to first estimate the upper plausible limit (UPL) followed by the lower plausible limit (LPL) so that they are 99% sure that the true survival probability was contained within that interval. Experts were also asked to estimate the most likely values (MLV). A web-based application was developed that would facilitate the elicitation and ensure experts were provided with immediate visual feedback regarding their elicitations, given that information at each time point was dependent on that in the previous time point. Before confirming each value, experts were challenged to consider whether they were certain about their estimates, in line with SHELF methodology. Following the individual expert elicitations, consensus about the appropriate long-term survival model from the perspective of a rational impartial observer was achieved in a follow-up meeting (which was the Gompertz model), which allowed experts to interact. A normal distribution was specified using each expert’s opinion about expected survival probabilities at each time point. The variance of this distribution was determined using the width of the interval provided by the expert. The survival beliefs of the experts implied interval-specific hazards that were incorporated with the ELIANA trial data. Posterior samples for the predicted survival from each expert were pooled to obtain the final survival distribution.

In our reanalysis, we consider the longer-term ELIANA data based on a median duration of follow-up of 24.2 mo (range: 4.5–35.1 mo)^9^ and combined this with the expert opinions for expected survival for years 4 and 5 (as we have an estimate of the survival function for times <2.8 y). We considered the expert beliefs at these time points and identified which distribution most accurately describes their beliefs, rather than assuming that they were normally distributed. We used the SHELF package to identify the best-fitting distribution to these time points by minimizing the least square error.^28^ Because we wished to include the expert’s MLVs, we modified these functions so that the MLV represented the mode of the distribution and included this quantity in the least squares optimization. The best fitting distribution was the one that minimized the sum of squares from either the normal, t, lognormal, gamma or beta distributions.

**Figure 2:**
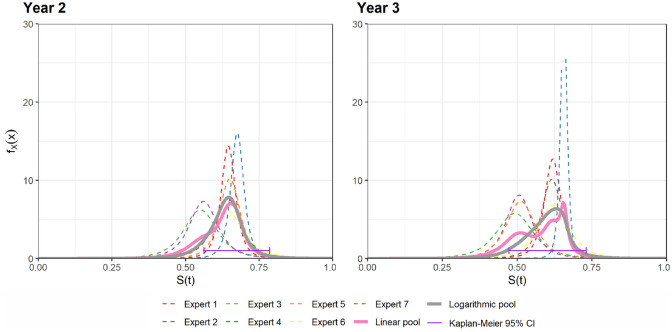
Experts’ opinion at years 2 and 3 including 95% Kaplan-Meier survival confidence intervals from updated ELIANA data at the same timepoints (purple interval).

Because we have updated data for survival at years 2 and 2.8 (which we assume is representative of year 3), it is important to confirm that the elicited survival at years 2 and 3 are broadly consistent with the survival at the same time points from the updated trial data. For consistency, we assumed the same distribution type for each expert across both years, so that the chosen distribution was the one that minimized the total sum of squares across years 2 and 3. The individual distributions for years 2 and 3 are presented in Figure 2. In addition, the logarithm and linear pooling and a purple interval representing the 95% Kaplan-Meier survival confidence intervals from updated ELIANA data at the same time points are plotted.

Figure 2 shows that the pooled distributions have more overlap with the 95% Kaplan-Meier survival confidence interval than the individual expert’s distributions and supports the finding that, in general, groups of experts tend to perform better than individuals.^24^ Although it is probable that experts would recalibrate their opinions on survival conditional on the longer-term follow-up, based on the observation that the pooled distributions for elicited survival at years 2 and 3 were similar to the follow-up data, it is reasonable to assume that these opinions remain valid, and we incorporate the year 4 and 5 opinions with the updated data.

**Figure 3:**
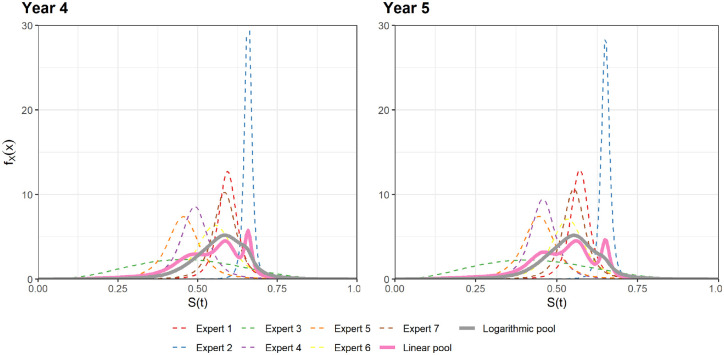
Best fitting distributions for experts’ opinions and aggregated distributions at years 4 and 5.

We repeated the approach described above for the expert opinions at years 4 and 5 with the individual and pooled distributions presented in Figure 3. Most of the expert beliefs were described by t distributions with 3 degrees of freedom. Expert 3’s opinion is best described using a beta distribution.

We see a variety of expert opinions, with Experts 1, 6, and 7 broadly similar, whereas Experts 4 and 5 are also similar. Across both time points, Expert 2 has a very strong opinion, while Expert 3 has a diffuse opinion. Although Expert 3 has the widest interval (UPL minus LPL), their MLV is also closer to the LPL, which results in the best-fitting distribution having a high standard deviation. Overall, this collection of opinions results in a trimodal distribution for the linear pool. The logarithm pool is smoother and assigns lower probability at the more extreme ends of the parameter space. Because linear pooling is the more common pooling method, we use the linear pooled distributions as representative of the expert opinions, which were then incorporated with the updated ELIANA trial data.

**Figure 4:**
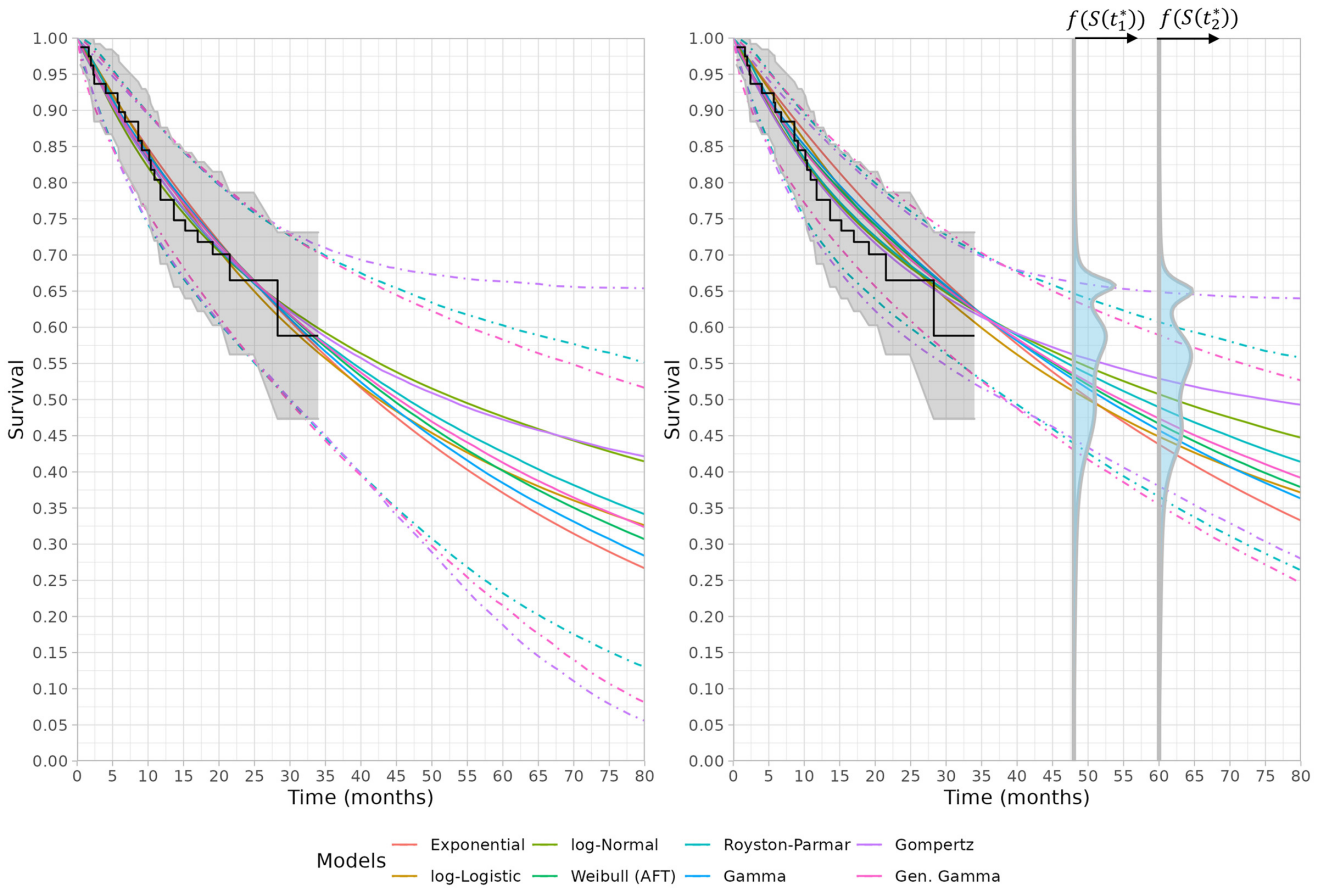
Left: Predicted survival functions fit using the updated ELIANA trial without expert opinion. Right: Predicted survival functions using the updated ELIANA trial with expert opinion at 48 and 60 months using linear pooling. The linear pooled distributions for the experts’ opinions are presented for both timepoints with f(S( $t_{1}^{*}$)) and f(S( $t_{2}^{*}$)) corresponding to (scaled) densities for the survival probability at the respective timepoints. 95% credible intervals are presented for the 3 models which have the largest change in 95% interval width at 60 months upon inclusion of expert opinion.

Figure 4 shows the predicted survival for the parametric models, including models using the data only (left) and the data together with expert opinion at years 4 and 5 (right). In addition to the posterior median survival for each model, the 2.5% and 97.5% quantiles are presented for the 3 models that have the largest change in 95% interval width at 60 mo (Gompertz, Royston-Parmar, and generalized gamma). For the models fit with Stan,^29^ inference was based on 3 chains each containing 10,000 iterations with the first 5,000 as burn-in, while for models fit with JAGS, each chain contained 50,000 iterations and the first 10,000 discarded as burn-in. As shown in Table 1, the log-logistic and log-normal models have the best statistical fit with respect to the deviance information criterion (DIC).^30^ Models that allowed for rapidly decreasing hazards (Gompertz) or nonmonotonic hazards (e.g., log-logistic or log-normal) seem to provide the best fit to the experts’ opinions and the data, a property that all of the three best-fitting models have. However, across all of the models considered, the differences in DIC are <3, suggesting that they are broadly similar in model fit. This is not surprising as the pooled prior is quite diffuse, with a 95% credible interval of 0.28–0.70 for the year 4 opinion and consequently predicted survival for all parametric models are plausible. If we estimate the models without the expert opinion, the exponential model, which assumes a constant hazard, was the best fit according to DIC. Including expert opinion assigns substantial probability to high long-term survival, and the parametric models that accommodate lower long-term hazards fit the data and expert opinion better.

**Table 1:** Survival Models Ordered by DIC for Expert Opinion Survival Models (Lower Is Better)

Model	DIC (Expert Opinion)	DIC (Vague Priors)
Log-logistic	273.81	277.65
Log-normal	273.83	278.32
Gompertz	274.02	278.53
Exponential	274.36	277.12
Generalized gamma	274.39	278.34
Weibull (AFT)	275.40	279.16
Royston-Parmar	275.52	280.02
Gamma	276.02	279.10

## Discussion

The primary contribution of this article is that it extends previous work on incorporating expert opinion to a wide range of parametric models.^5,17^ In contrast to previous work, the introduced approach makes it straightforward to incorporate information about other quantities of interest (e.g., median, mean survival, or mean survival difference) into an analysis. In addition, this article highlights important considerations with respect to pooling information from multiple experts. Specifically, we describe the estimation of the best-fitting probability distributions to each individual opinions, the differences in 2 pooling approaches, and how multimodal aggregated distributions can be incorporated into the analysis. Our approach permits the use of model selection criteria such as the DIC, so that models that have incorporated expert opinion can be objectively compared.

Our analysis of the ELIANA trial data results in similar conclusions as the analysis performed by Cope et al.^5^ In their analysis, the preferred model was the Gompertz, whereas in ours, the log-logistic ranked highest, with both models implying decreasing hazards. In the approach presented by Cope et al.,^5^ estimates from each expert were modeled separately, and the overall estimate reflected a combined overall distribution. This necessitated fitting models for each of the individual experts before combining the results. The authors noted that this approach avoids pooling or model averaging, which would provide narrower intervals around the mean. We argue that such an approach does not avoid pooling and is actually a linear pool of the posterior distributions. As our illustrative example in the section on incorporating multiple expert opinions shows, this does not automatically lead to narrower intervals. Decision makers may value an aggregated prior, as it aids understanding about how the prior changes the analysis, compared with an analysis using the data alone.

We generally prefer the outcome that we are eliciting to be a single probability distribution representing the combined knowledge of experts in the field.^31^ Resolving the experts’ judgments into a single distribution is known as the problem of aggregation. In this article, we use mathematical aggregation (as we do not have access to the experts); however, we note that the SHELF framework permits behavioral aggregation in which the group of experts discuss their knowledge and opinions to form “consensus” judgments, to which an aggregate distribution is agreed. Even in situations in which behavioral aggregation is the objective, using a mathematical aggregation of the experts’ opinions may be a useful visual tool in agreeing with the consensus distribution. Although expert opinion can be of value in reducing the differences in extrapolated survival probabilities for different parametric models, the appropriate elicitation of these quantities is challenging. One important point is how the questions are framed, with Bousquet providing examples of some open questions that are relevant when eliciting beliefs about survival.^13^ Clearly defined elicitation questions are particularly relevant, as the experts may not be familiar with statistical terms and can misinterpret averages as medians.^13^ It has also been frequently discussed that experts can be overconfident^13,31,32^ and that calibration and differential weighting of experts may reduce this overconfidence.^32^ Within this analysis, it is possible that Expert 2 provided survival estimates that were overconfident, and exclusion of this expert’s opinion slightly lowers the expected survival estimates, although the ordering of DIC for the parametric models remains broadly the same, with the Gompertz, log-normal, and log-logistic remaining the top 3 models. When considering the pooled distributions, the 95% intervals of the expert opinions at years 2 and 3 were similar to the 95% intervals from the Kaplan-Meier curves at years 2 and 3 for the updated ELIANA data, suggesting that it is appropriate to incorporate the pooled information at years 4 and 5 into our analysis. Because all of the experts had extensive experience in using tisagenlecleucel (or related treatments) in the target population, their pooled opinions can be considered more robust than relying exclusively on the short-term trial data. When the pooled expert opinion was incorporated into the survival analysis, this led to reduced uncertainty in the resultant survival projections. As shown in Figure 4, the 95% survival credible intervals for each of the survival models lie within the 95% credible intervals of the expert linear pooled distribution at years 4 and 5.

Using model fit statistics will provide only an assessment of fit to the observed data, and a final decision on the choice of model should also be based on clinical plausibility. Incorporating expert opinion in methodologically appropriate ways is therefore a robust way to ensure that decision makers have plausible evidence available to them. Often the plausibility of a parametric model is assessed on the basis that the predicted survival is within an appropriate survival probability interval at a number of landmark time points (i.e., between 20%–40% at year 5 and 10%–20% at year 10). In our opinion, it is best practice to incorporate this information explicitly, and our approach allows for the direct synthesis of these beliefs with the observed data. This approach would be particularly useful in situations in which none of the available model projections are considered plausible by decision makers when using data alone, due to, for example, data immaturity or differences in standards of clinical practice in different countries. If reliable expert opinion is available and can be elicited, our approach permits recalibration of these models to more accurately reflect the survival projection of the population of interest.

Because the opinions elicited from the expert (and parameterized as probability distributions) will almost surely not be centered on the true parameter value (i.e., true survival at a time point), it is worth considering for which situations including an expert opinion will lead to better estimates of extrapolated survival than using data alone. We explore this in Appendix D through a simulation study and find that, in general, if the expert under- or overestimates the true survival by ≤25% (in relative terms), including an expert opinion provides better estimates than using data alone, assuming both the parametric model and data-generating process are the same (both Weibull). In the situation in which the parametric model chosen was a log-normal and the data-generating process was a Weibull distribution, the inclusion of expert opinion produced better extrapolations even when the expert underestimated survival by 40%. We believe that the inclusion of expert opinion can make extrapolation of survival outcomes more reliable and robust to misspecification of the parametric model. Owing to the number of factors that affect extrapolated survival, further research in this topic is needed.

Although not discussed in this article, there are situations in which the expert may have considerable experience with the comparator arm and may be more comfortable providing an opinion on the plausible survival probabilities for the comparator at particular time point(s). If a relationship such as proportional hazards (PH) or accelerated time factor (ATF) can be considered tenable (i.e., evaluated based on trial data and assumed to hold in the long term), a survival model with the PH or ATF parameterization with treatment status as a covariate could be estimated. Alternatively, experts may be willing to provide an estimate of the expected survival difference between 2 treatments. Both of these approaches have been implemented, and we provide simulated examples of each situation in the expertsurv package available on GitHub.

As noted in the third section, similar results can be obtained using frequentist methods (although this would not be the case for the multimodal priors in the fourth section), and expertsurv provides code based on the flexsurv package to accommodate this.^20^ Although the incorporation of expert opinion is relatively straightforward with the approach described in this article, further research on elicitation of long-term survival probabilities and best practices are important if expert opinions are to become more widely used in health technology assessments using time-to-event outcomes.

## Supplemental Material

sj-docx-1-mdm-10.1177_0272989X221150212 – Supplemental material for Direct Incorporation of Expert Opinion into Parametric Survival Models to Inform Survival Extrapolation Click here for additional data file. Supplemental material, sj-docx-1-mdm-10.1177_0272989X221150212 for Direct Incorporation of Expert Opinion into Parametric Survival Models to Inform Survival Extrapolation by Philip Cooney and Arthur White in Medical Decision Making

## Notes

a. This is true if the expert opinion has a closed form expression. For example, the Royston Palmer spline models do not have such an expression for the mean survival. Although we could derive this with manual integration, it would be computationally intensive to do so at each step in the MCMC algorithm.

## References

[1] Rutherford MJ Lambert PC Sweeting MJ , et al. Flexible Methods for Survival Analysis TSD. London: National Institute for Health and Care Excellence; 2020.

[2] Monnickendam G Zhu M McKendrick J , et al. Measuring survival benefit in health technology assessment in the presence of nonproportional hazards. Value Health. 2019;22:431–8. 10.1016/j.jval.2019.01.005 30975394

[3] Davies C Briggs A Lorgelly P , et al. The “hazards” of extrapolating survival curves. Med Decis Making. 2013;33:369–80. 10.1177/0272989X12475091 23457025

[4] Jackson CH Sharples LD Thompson SG . Survival models in health economic evaluations: balancing fit and parsimony to improve prediction. Int J Biostat. 2010;6(1):Article 34. 10.2202/1557-4679.1269 21969987

[5] Cope S Ayers D Zhang J , et al. Integrating expert opinion with clinical trial data to extrapolate long-term survival: a case study of CAR-T therapy for children and young adults with relapsed or refractory acute lymphoblastic leukemia. BMC Med Res Methodol. 2019;19:182. 31477025 10.1186/s12874-019-0823-8 PMC6721254

[6] Guyot P Ades AE Beasley M , et al. Extrapolation of survival curves from cancer trials using external information. Med Decis Making. 2017;37:353–66. 10.1177/0272989X16670604 PMC6190619 27681990

[7] O’Hagan A Buck CE Daneshkhah AE , et al. Multiple experts. In: Uncertain Judgements: Eliciting Experts’ Probabilities. New York: John Wiley & Sons; 2006. p 179–92.

[8] Grupp S Laetsch T Buechner J , et al. Analysis of a global registration trial of the efficacy and safety of CTL019 in pediatric and young adults with relapsed/refractory acute lymphoblastic leukemia (ALL). Blood. 2016;128:221.

[9] Grupp SA Maude SL Rives S , et al. Updated analysis of the efficacy and safety of tisagenlecleucel in pediatric and young adult patients with relapsed/refractory (r/r) acute lymphoblastic leukemia. Blood. 2018;132:895.

[10] R Core Team. R: A Language and Environment for Statistical Computing. 2021. Available from: https://www.R-project.org/

[11] Campodonico S Singpurwalla N . Expert Opinion in Reliability. Washington DC: George Washington University, Institute for Reliability and Risk Analysis; 1993.

[12] Singpurewalla ND Song MS . Reliability analysis using Weibull lifetime data and expert opinion. IEEE Transact Reliabil. 1988;37:340–7.

[13] Bousquet N . A Bayesian analysis of Industrial Lifetime Data with Weibull Distributions [Research Report] RR-6025. Le Chesnay-Rocquencourt (France): INRIA; 2006.

[14] Coolen FPA . On Bayesian reliability analysis with informative priors and censoring. Reliabil Eng Syst Safety. 1996;53:91–8.

[15] Bedrick EJ Christensen R Johnson W . A new perspective on priors for generalized linear models. J Am Stat Assoc. 1996;91:1450–60.

[16] Johnson Wesley O . Predictive influence in the log normal survival model. In: Lee JC Johnson WO Zellner A, eds. Modelling and Prediction Honoring Seymour Geisser. New York: Springer; 1996.

[17] Ouwens M . Use of Clinical Opinion in the Estimation of Survival Extrapolation Distributions. Lawrenceville (NJ): ISPOR; 2018.

[18] Wongnak P Bord S Donnet S , et al. A hierarchical Bayesian approach for incorporating expert opinions into parametric survival models: a case study of female Ixodes ricinus ticks exposed to various temperature and relative humidity conditions. Ecol Model. 2022;464:109821.

[19] Plummer M . JAGS: A program for analysis of Bayesian graphical models using Gibbs sampling. In: Proceedings of the 3rd International Workshop on Distributed Statistical Computing (DSC 2003); 20–22 March 2003; Vienna, Austria.

[20] Jackson C . flexsurv: A platform for parametric survival modeling in R. J Stat Softw. 2016;70:1–33. 10.18637/jss.v070.i08 PMC5868723 29593450

[21] Bissiri PG Holmes CC Walker SG . A general framework for updating belief distributions. J R Stat Soc Ser B (Stat Methodol). 2016;78:1103–30. 10.1111/rssb.12158 PMC5082587 27840585

[22] Cole S Chu H Greenland S . Maximum likelihood, profile likelihood, and penalized likelihood: a primer. Am J Epidemiol. 2013;179:252–60. 10.1093/aje/kwt245 PMC3873110 24173548

[23] Nocedal J Wright S . Numerical Optimization. New York: Springer Science & Business Media; 2006.

[24] Clemen RT Winkler RL . Combining probability distributions from experts in risk analysis. Risk Anal. 1999;19:187–203. 10.1111/0272-4332.202015 10859775

[25] Salvo FD . A characterization of the distribution of a weighted sum of Gamma variables through multiple hypergeometric functions. Integral Transforms and Special Functions. 2008;19:563–75.

[26] Grupp SA Maude SL Shaw PA , et al. Durable remissions in children with relapsed/refractory ALL treated with T cells engineered with a CD19-targeted chimeric antigen receptor (CTL019). Blood. 2015;126:681.

[27] Maude SL Pulsipher MA Boyer MW , et al. Efficacy and safety of CTL019 in the first US phase II multicenter trial in pediatric relapsed/refractory acute lymphoblastic leukemia: results of an interim analysis. Blood. 2016;128:2801.

[28] Oakley J . SHELF: Tools to Support the Sheffield Elicitation Framework. City (ST): Publisher; 2020.

[29] Stan Development Team. RStan: The R Interface to Stan. City (ST): Publisher; 2020.

[30] Spiegelhalter DJ Best NG Carlin BP , et al. Bayesian measures of model complexity and fit. J R Stat Soc Ser B (Stat Methodol). 2002;64:583–639.

[31] O’Hagan A . Expert knowledge elicitation: subjective but scientific. Am Stat. 2019;73:69–81.

[32] Lin S-W Bier V . A study of expert overconfidence. Reliabil Eng Syst Safety. 2008;93:711–21.

