# Supplementary material for: Direct Incorporation of Expert Opinion into Parametric Survival Models to Inform Survival Extrapolation
Source: Med Decis Making. 2023 Jan 16;43(3):325–36. doi: 10.1177/0272989X221150212 (PMC10021125; doi:10.1177/0272989X221150212)
Supplement: sj-docx-1-mdm-10.1177_0272989X221150212 – Supplemental material for Direct Incorporation of Expert Opinion into Parametric Survival Models to Inform Survival Extrapolation [file sj-docx-1-mdm-10.1177_0272989X221150212.docx]

# Appendix A Validation of approach

We compare the results of our method to a previous publication.^12^ They consider a Weibull distribution with a proportional hazards (PH) parametrization and incorporate expert opinion on the median survival as described below. The median survival time is $t_{0.5}=\frac{{\log(2)}^{1/a}}{m}=\kappa$. Re-expressing the distribution in terms of $\kappa$, they obtain survival function $S\left( t \right)=\exp\left\{ -log(2)\left( \frac{t}{\kappa} \right)^{a} \right\}$ and hazard function $h\left( t \right)= \frac{\log\left( 2 \right)at^{a-1}}{\kappa^{a}}.$ The expert belief about $\kappa$ is characterized by the location or mean $l$ and standard deviation $s$. It is also possible to consider additional parameters $c$ and $v$ which can be used to calibrate the expert's opinion about $l$ and $s$, however, in the case where the analyst does not wish to modulate the expert's opinion then $c=1$ and $v= \frac{1}{2}$. Under some mild assumptions $\left[ \frac{c^{2}v}{sl^{2}} \right]\kappa^{2}\approx\chi^{2}(\frac{v}{s}+1)$ and assuming no calibration of the expert's opinion and noting that the square root of a $\chi^{2}\left( n \right)$ random variable is a $\chi(n)$ random variable $\left[ 1/\sqrt{2s}l \right]\kappa\approx\chi\left( \left( \frac{v}{s} \right)+1 \right)$. Therefore $\kappa$ is a $\chi(\frac{v}{s}+1)$ random variable scaled by $\sqrt{2s}\times l$and if we assume that $a\sim G\left( \alpha, \beta\right)$ which are elicited from the expert then both parameters are specified.

In the original publication a Bayes estimator was derived for the parameters using some approximations; however, it is straightforward to use JAGS or Stan to obtain the complete posterior distribution. Using simulated data they provide in the paper, they set $l=500, s=200, \alpha=6.25$ and $\beta=12.5$ and did not assume any modulation of the expert's opinion. Using the same chi-squared prior for the median survival we also estimate the model using our approach. This gives a prior for the median survival (termed Original Prior) in Figure 5 and posterior survival curves in Figure 6a. The fact that the posterior distributions are very similar to the analysis without any expert opinion is unsurprising as the original prior had a significant probability within the 95% confidence interval implied by the data alone (8909 - 22188). In a second example we adjust the prior belief of the expert to yield a much lower median value (termed adjusted prior in Figure 5) and see that the mean survival posterior for both approaches incorporating expert opinion are very similar and as expected, outside the confidence interval for the median (see Figure 6b). This highlights that our proposed approach is consistent with previous methods.

Figure 5: Original prior and adjusted prior for median survival


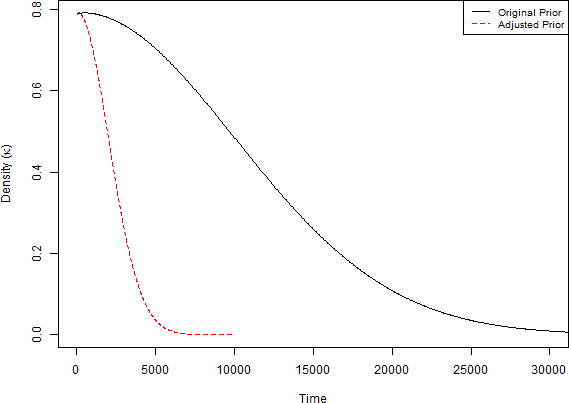


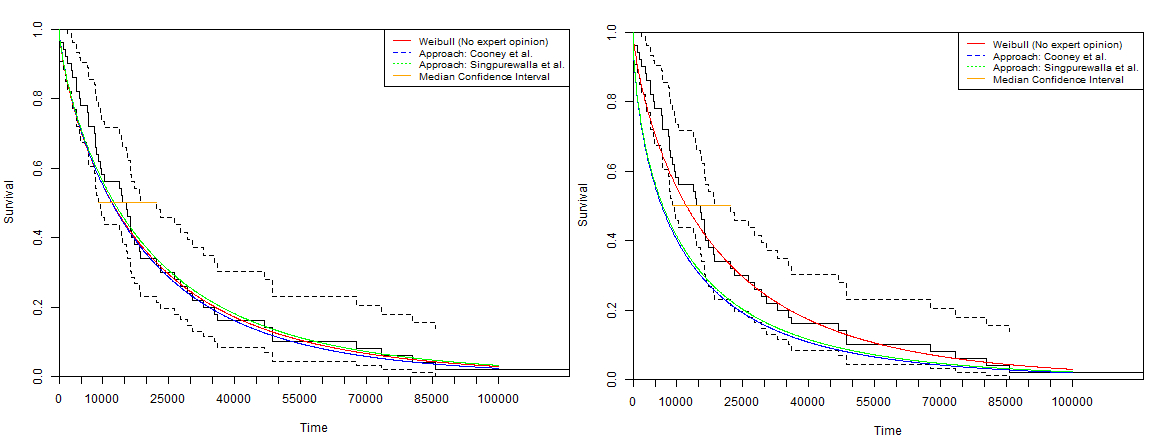


Figure 6: (a) Survival functions under the original prior (b) Survival functions under the adjusted prior

# Software

All methods outlined in this paper are available for use at https://github.com/philip-cooney/expertsurv. The code in the package is adapted from the package survHE,^33^ which primarily considers survival analysis from a Bayesian approach, and flexsurv, which provides a frequentist implementation.^20^ We allow a user to supply opinions on survival timepoints and expected differences with worked examples provided in the README file. For the purposes of comparison, we also show how the same functions can be used to fit the models without expert opinion.

# Appendix B Technical Details for models fit in JAGS

We could not fit the gamma, Gompertz and generalized gamma models in Stan and we instead fit these models in JAGS. We describe how we analytically evaluate the expected survival for the Gompertz and generalized gamma distributions.

The expectation of a Gompertz distributed random variable with shape parameter $a^{*}=\frac{1}{b}$and rate parameter $b^{*}=a/b$ is $E\left[ T \right]=b\text{exp}\left( a \right)E_{a}(-1)$ where $E_{a}\left( t \right)=\int_{1}^{\infty} u^{t}\text{exp}\left( -au \right)du$ (where $a^{*},b^{*}$are flexsurv parameterizations).^34^ We note that $E_{a}\left( t \right)=\Gamma\left( 0,a \right)$, the upper incomplete gamma function.^35^ We need to approximate this function, and note that by definition the gamma function $\Gamma(x$) is the sum of the upper $\Gamma(x,a)$ and lower $\gamma(x,a)$ incomplete gamma functions $\Gamma\left( x \right)=\Gamma\left( x,a \right)+ \gamma(x,a)$. Hence, $\Gamma\left( 0,a \right)= \lim_{x\to0} \Gamma\left( x \right)-\gamma(x,a)$where for practical purposes we set x = 0.0001. By definition, we can compute the lower incomplete gamma function as a product of the gamma function and the cumulative distribution function of the gamma distribution $\Gamma\left( x \right)F\left( x=a; \alpha=x,\beta=1 \right).$We finally have the expected survival as $\frac{1}{a^{*}}\text{exp}\left\{ \frac{b^{*}}{a^{*}} \right\}\Gamma\left( 0,\frac{b^{*}}{a^{*}} \right).$

For generalized gamma, the parameterization in JAGS is slightly different to the original parameterization with $b\times r=d$, $b=p$ and $\lambda=\frac{1}{a}$, which gives the mean as $\Gamma\left( (b\times r+1)/b \right)/(\lambda\Gamma\left( r \right))$.^36^ For consistency of results with the flexsurv package we have $\mu= -\log\left( \lambda\right)+\frac{\log\left( r \right)}{b}$, $\sigma=1/(b\sqrt{r})$ and $Q=\sqrt{\frac{1}{r}}$.

# Appendix C Simulation study - Effect of priors on posterior survival when including expert opinion

As noted in the main text we wished to assess if the weakly informative priors typically used in Bayesian analysis could conflict with the information provided by the expert. To investigate this, we conducted a simulation study comparing the posterior survival of the models with expert opinion under two specifications of weakly informative priors; one in which the priors for all parameters were uniform and alternatively where the priors for parameters had normal or gamma distributions (the latter for parameters which are constrained to be positive). In addition to the priors having a different parametric form, a further difference was that the standard deviations for normal and gamma distributions were relatively low for weakly informative priors. For example, the log of the scale parameter for the Weibull model was a normal distribution with mean 0 and standard deviation 1 and the shape parameter was a gamma distribution with both parameters equal to 1. This is in contrast to the standard deviations of the uniform priors, which were typically >28.

In the simulation study we generated data from a Weibull (proportional hazards) model for a variety of parameters and sample sizes (n = 30,50,100) with a maximum follow-up of 2 years. We incorporated different values of expert opinion in terms of mean and standard deviation at multiple timepoints, assuming that the expert’s opinion was a normal distribution. Taking all combinations of the parameters described in Table 2 produced 324 simulations across each of the parametric models.

Table 2: Parameters used in Simulation Study

| **Parameter** | **Values** |
| --- | --- |
| Shape | 0.75, 1, 1.25 |
| Scale | 0.25,0.5,0.75, 1 |
| Sample Size | 30,50,100 |
| Mean value of S($t_{1}^{*}$) | 0.1, 0.3 |
| Mean value of S($t_{2}^{*}$) | 0.05, 0.1 |
| SD of expert’s opinion† | 0.025, 0.05, 0.1 |

S($t_{1}^{*}$) S($t_{2}^{*}$) denotes the survival at 4 and 10 years – only evaluated scenarios in which S($t_{1}^{*}$)>S($t_{2}^{*}$)

† Standard deviation (SD) of the expert opinion was equal across both timepoints

To assess the similarity of the survival curves under each prior specification we evaluated the posterior median restricted mean survival time (RMST) until a timepoint of 15 years for each model. To provide a measure of similarity on a comparable scale, we estimated the ratio $\frac{min(RMST_{uniform},RMST_{non-uniform})}{max(MST_{uniform},RMST_{non-uniform})}$, with $RMST_{uniform}$ and $RMST_{non-uniform}$ denoting the RMST under each prior specification. Overall, all models had very high RMST ratio values with median values of 0.99 even at a sample size of 30 as shown in Table 3. Results for larger sample sizes were even larger.

A simulation study using the same specifications as described in Table 2 compared the Bayesian approach (with uniform priors) to the estimates derived from the penalized maximum likelihood approach (which does not require the specification of a prior). If the distribution representing the expert opinion was not multi-modal, the ratios of RMST were very close to 1 even at sample sizes of 30. Based on these results it can be concluded that weakly informative prior distributions do not conflict with the information provided by the expert.

Table 3: RSMT ratios for each parametric model with dataset of 30 observations in both simulation studies

| **Model** | **Median RMST Ratio – Uniform vs Normal/Gamma Priors** | **Median RMST Ratio – Uniform vs Penalized Maximum Likelihood Estimates** |
| --- | --- | --- |
| Exponential | 0.99 | 0.99 |
| Weibull | 0.99 | 0.97 |
| log-Logistic | 0.99 | 1 |
| log-Normal | 0.99 | 0.99 |
| Royston-Parmar | 1 | 0.98 |
| Gompertz | 0.99 | 0.97 |
| Gamma | 0.99 | 0.97 |
| Gen. Gamma | 0.99 | 0.98 |

For illustration, Figure 7 below shows examples of the posterior survival functions (along with 95% intervals as dashed lines) for the Gompertz, Weibull, Royston-Parmar spline model and generalized gamma for scenarios in which the sample size was 30 observations. The RMST ratio is provided in the title of each plot. Expert opinion is indicated by the dashed vertical lines at times 4 and 10 and includes situations where the mean values of S($t^{*}$) are both high and low and also informative and vague (small and large standard deviations).


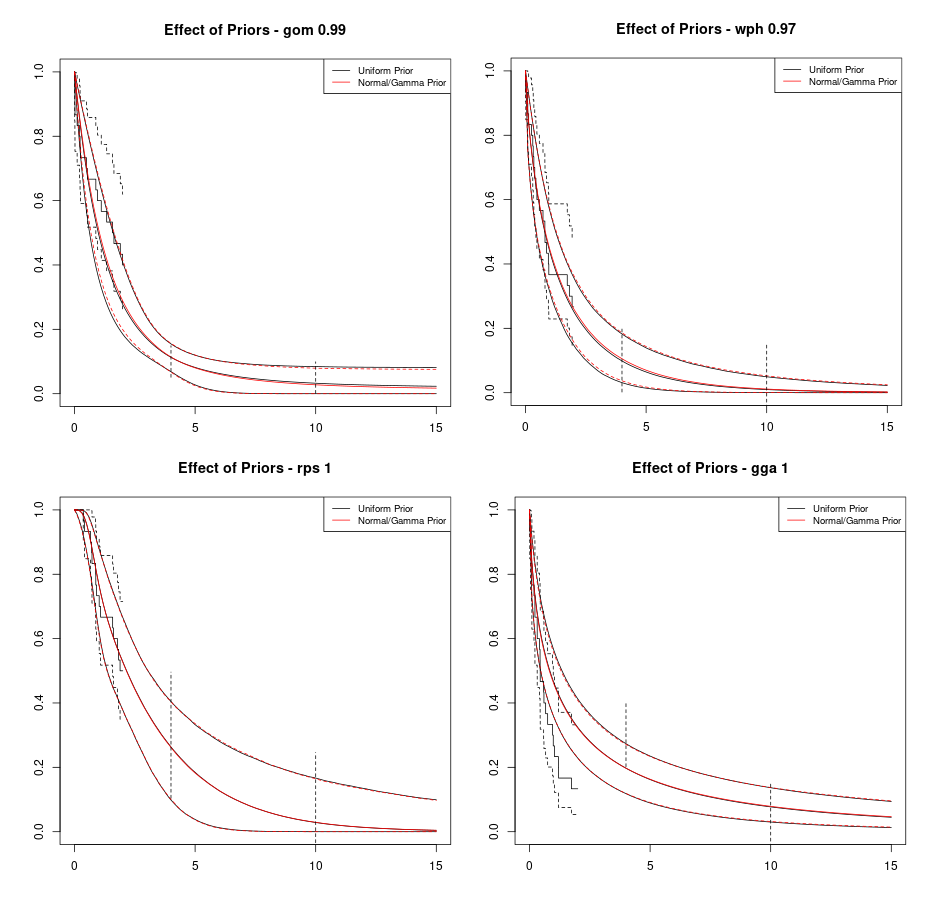


Figure 7: Survival curves when under different specifications of minimally informative priors

Similar illustrations are provided for the comparison between the Bayesian models with expert opinion and the penalized maximum likelihood approach for gamma, log-normal, Royston-Parmar and Weibull models (Figure 8). Also included for reference are the Bayesian and maximum likelihood models without any expert opinion.


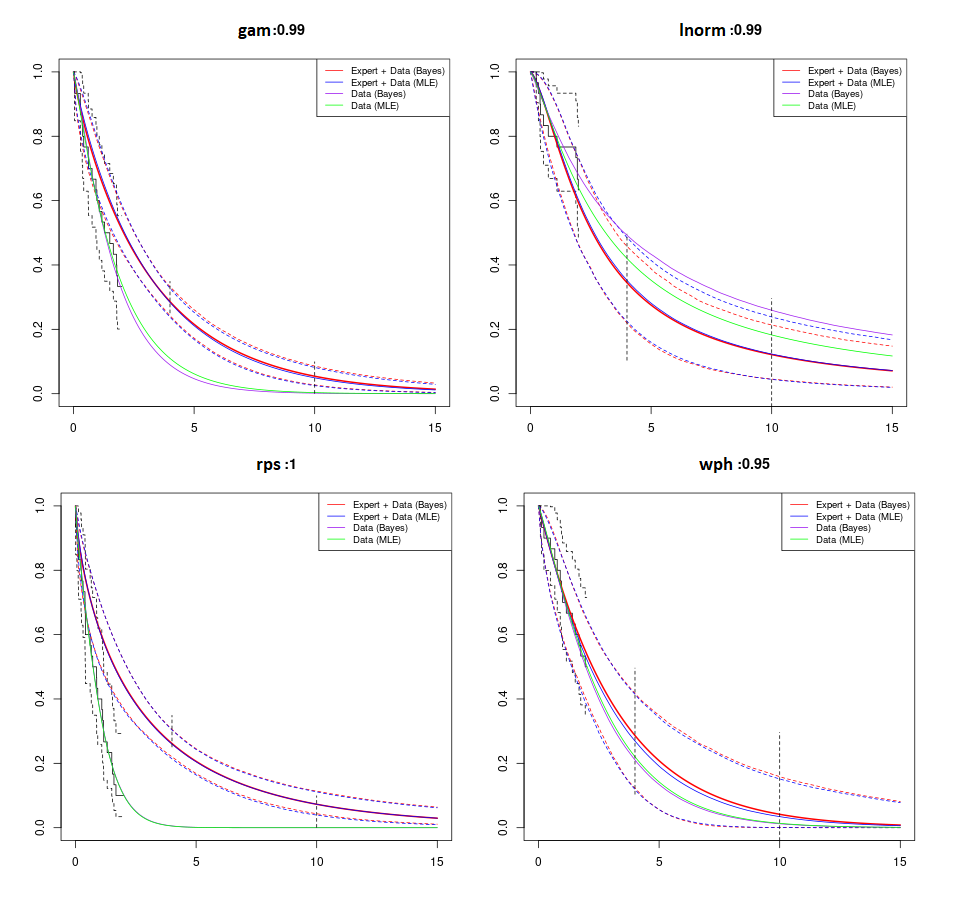


Figure 8: Survival curves with and without expert opinion for both Bayesian and (penalized) maximum likelihood approaches

# Appendix D Simulation study – Impact of Bias in Expert Opinion on Extrapolated Survival

As noted in main text, it is almost certain that the opinions elicited from the expert (and parameterized as probability distributions) will not be centred on the true value. Considering beliefs elicited about survival probabilities at timepoints, if the expected value of an expert’s opinion is different from the true survival at a particular timepoint, then the expert’s opinion is biased relative to the true survival. However, in many situations it can still be closer (on average) to the true survival function than using the data alone.

To make this statement more concrete, consider an example in which 30 observations are generated by a Weibull probability distribution with shape equal 1, scale equal 0.1 (proportional hazards parameterization) and a maximum follow up time of 2 years. If the expert assumes that their belief about survival at 10 years is characterized by a normal distribution with a mean of 0.46 and standard deviation of 0.05, the expected value of their opinion is 25% above the true value of 0.367, i.e. biased by a factor of 1.25.

If we fit a Weibull model to the data, the maximum likelihood estimate will (on average) provide an approximately unbiased survival curve, however, because we have a limited sample size and do not observe data after 2 years the estimate is associated with a significant degree of uncertainty. In contrast, the survival curve (at the restricted maximum likelihood estimate^[[1]](#footnote-1)^) obtained from including the expert’s opinion will be biased but have a considerable reduction in uncertainty and will be on average closer to the true survival function. To produce stable results, 500 datasets are simulated under the conditions described above and models fit by (restricted) maximum likelihood including and excluding the expert’s opinion. Figure 9 presents the median (solid line) and 95% quantiles (dashed lines) of the survival estimated with (purple) and without (red) expert opinion. To be clear, these are the quantiles of the survival over the 500 datasets at the restricted and regular maximum likelihood estimates and do not refer to the confidence intervals from a given model.


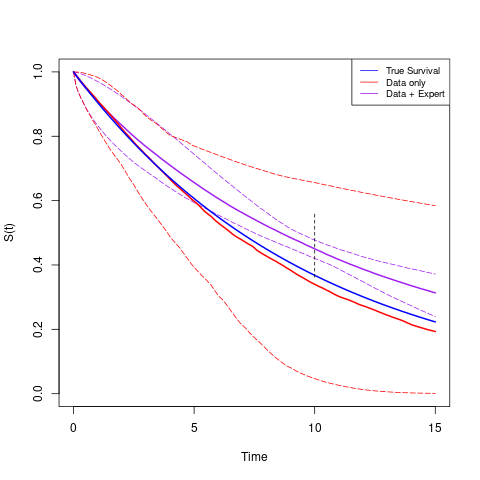


Figure 9: Estimation of expected survival functions with and without expert opinion

From Figure 9 we get a sense of the bias-variance trade off. Although the survival at 10 years estimated by the penalized maximum likelihood estimators are always above the true survival for each of the 500 datasets (ranging from 0.42-0.48), they are less than 0.11 from the true value in 97.5% of the datasets. In contrast when using the data alone the 95% interval for the survival is 0.04-0.66 meaning that in 5% of the datasets either underestimated the survival by $\geq$0.32 or overestimated it by $\geq$0.29.

In order to get a numerical estimate of the bias-variance trade off, we estimated the mean squared error (MSE) for the restricted mean survival time (RMST), up to 15 years: $MSE= {(RMST-\hat{RMST})}^{2}$with $RMST$ referring to the true value and $\hat{RMST}$ the estimate based on the models, for both the model with and without expert opinion (i.e. data alone) in each of the 500 simulations. To get a single number to compare the results between both models, we evaluated the mean of the difference (across the 500 simulations) between the MSE from the model without expert opinion and MSE with expert opinion. Values >0 mean that on average the MSE for the model with expert opinion was lower than that without expert opinion.

In our example the median MSE was 5 without using expert opinion, and 0.94 using expert opinion. Across the 500 simulations the average difference of MSE without expert opinion against MSE with expert opinion was 5.55. We also evaluated the absolute difference of the RMST, $|RMST-\hat{RMST}|$ for both the models with and without expert opinion and evaluated the mean difference. Using this measure (the absolute deviation rather than squared deviation) places a lower penalty than MSE on having values further away from the true value. In this situation the expected difference in absolute difference of RMST was 1.22, considerably lower than the equivalent value based on the squared deviation but nevertheless substantial, as the true RMST was 7.77.

We expect a variety of parameters to influence the MSE with and without expert opinion, in particular degree of bias, strength of belief (as indicated by the standard deviation) and the sample size. These factors and others relating to the follow-up time, parameters for the Weibull distribution and timepoints of expert’s opinion (parameterized as a normal distribution) are presented in Table 4. We considered specifications based on each of the combinations of parameters, to yield 576 specifications.

Table 4: Parameters for simulation study

| **Parameter** | **Values** |
| --- | --- |
| Shape | 0.75, 1, 1.25 |
| Scale | 0.25,0.5,0.75, 1 |
| Sample Size | 30,50,100 |
| Standard Deviation of expert’s opinion* | 0.1,0.05,0.025 |
| Timepoints for expert opinion | 4 only, 10 only, 4 and 10 |
| Longest follow up time (i.e. observations after this assumed censored)* | 2, 4 |
| Bias factor of expert – factor by which the expert under/overestimates true survival | 0.6,.75, 1.25 |

* If follow up time was 4 years, then expert opinion was only incorporated at 10 years

Overall, only 70 of the 576 (12%) simulations had an expected difference in MSE < 0, denoting that on average the MSE with expert opinion was worse than without expert opinion. In the case of absolute deviation this number increased to 118 simulations (20%). Considering the results summarized by bias factor of expert and standard deviation of expert opinion (Table 5), the percentage of scenarios in which (expected) absolute deviation from RMST was lower with expert opinion was above 70% for all scenarios in which the bias factor of the expert was between 0.75-1.25 and suggests that expert opinions within this range improve the prediction of long term survival outcomes.

Perhaps unsurprisingly the situations where the expert opinion had a higher (worse) MSE included when the expert was biased and the true survival was quite high i.e. above 40% for years 4 or 10. This is because we included a relative bias, assuming the survival is 1.25 times the true survival at 50% results in a greater absolute error than when it is only 25%. From the perspective of including expert opinion, the worst results were obtained for bias factors farther away from 1, large sample sizes, and in which the confidence of the expert was high e.g. standard deviation equal to 0.025. Lower MSE was achieved in situations where expert opinion was incorporated at the 10 year timepoint rather than the 4 year timepoint.

Table 5: Percentage of scenarios in which model with expert opinion performed better than model based on data alone (Models and data assumed to be from a Weibull distribution)

| **Bias factor of expert** | **SD of expert’s opinion** | **% Scenarios in which (average) squared deviation from RMST was lower with expert opinion** | **% Scenarios in which (average) absolute deviation from RMST was lower with expert opinion** | **Number of Scenarios** |
| --- | --- | --- | --- | --- |
| 0.6 | 0.025 | 60% | 40% | 48 |
| 0.6 | 0.05 | 67% | 50% | 48 |
| 0.6 | 0.1 | 85% | 65% | 48 |
| 0.75 | 0.025 | 85% | 75% | 48 |
| 0.75 | 0.05 | 90% | 81% | 48 |
| 0.75 | 0.1 | 96% | 94% | 48 |
| 0.9 | 0.025 | 100% | 100% | 48 |
| 0.9 | 0.05 | 100% | 100% | 48 |
| 0.9 | 0.1 | 100% | 100% | 48 |
| 1.25 | 0.025 | 83% | 73% | 48 |
| 1.25 | 0.05 | 88% | 77% | 48 |
| 1.25 | 0.1 | 100% | 100% | 48 |

SD - Standard Deviation

It is also worth highlighting that results of the simulation study presented in Table 5 was based on the assumption that the true model, a Weibull distribution, was selected. We repeated the simulation study assuming that the data were still generated by a Weibull distribution but that a log-normal distribution, i.e., an incorrect parametric model was fit to the data instead. Table 6 shows that for all combinations of bias and standard deviation of expert’s opinion, the (expected) absolute deviation from RMST was lower with expert opinion in more than 70% of scenarios, highlighting that the inclusion of expert opinion can make extrapolation of survival outcomes more robust to misspecification of the parametric model.

Table 6: Percentage of scenarios in which log-normal model with expert opinion performed better than log-normal model based on data alone assuming data was generated by a Weibull distribution

| **Bias factor of expert** | **SD of expert’s opinion** | **% Scenarios in which (average) squared deviation from RMST was lower with expert opinion** | **% Scenarios in which (average) absolute deviation from RMST was lower with expert opinion** | **Number of Scenarios** |
| --- | --- | --- | --- | --- |
| 0.6 | 0.025 | 81% | 77% | 48 |
| 0.6 | 0.05 | 94% | 83% | 48 |
| 0.6 | 0.1 | 100% | 100% | 48 |
| 0.75 | 0.025 | 100% | 100% | 48 |
| 0.75 | 0.05 | 100% | 100% | 48 |
| 0.75 | 0.1 | 100% | 100% | 48 |
| 0.9 | 0.025 | 100% | 100% | 48 |
| 0.9 | 0.05 | 100% | 100% | 48 |
| 0.9 | 0.1 | 100% | 100% | 48 |
| 1.25 | 0.025 | 81% | 81% | 48 |
| 1.25 | 0.05 | 81% | 81% | 48 |
| 1.25 | 0.1 | 83% | 81% | 48 |

SD - Standard Deviation

**References**

33. Baio G. survHE: survival analysis for health economic evaluation and cost-effectiveness modeling. J Stat Softw. 2020;95:1–47.

34. Siegrist K. The Gompertz distribution. 2021. Available from: <http://www.randomservices.org/random/special/Gompertz.html>

35. Weisstein EW. MathWorld—exponential integral. 2021. Available from: https://mathworld.wolfram.com/ExponentialIntegral.html

36. Stacy EW. A generalization of the gamma distribution. Ann Math Stat. 1962;33:1187–92.

1. We use the (penalized) maximum likelihood approach as we must fit the model a large number of times for each specification of the parameters. [↑](#footnote-ref-1)
